# Descriptive Rules for Achalasia of the Esophagus, June 2012: 4th Edition

**DOI:** 10.1007/s10388-017-0589-1

**Published:** 2017-09-05

**Authors:** Japan Esophageal Society

**Affiliations:** Hirose-Building 4F, Taihei 2-3-13, Sumida-ku, Tokyo, 130-0012 Japan

Review Committee for Descriptive Rules for Achalasia of the Esophagus

*President*

Hisahiro Matsubara

*Former President*

Nobutoshi Ando

*English Edition Committee, Chairman*

Nobuo Omura

*Adviser*

Hideyuki Kashiwagi

*English Edition Committee Members*

Soji Ozawa: Surgery
Tatsuyuki Kawano: Surgery
Hiroyasu Makuuchi: Surgery
Katsuhiko Iwakiri: Internal Medicine
Motoyasu Kusano: Internal Medicine
Ken Haruma: Internal Medicine
Kaiyo Takubo: Pathology
Akio Yanagisawa: Pathology

*Japanese Edition Committee, Chairman*

Hideyuki Kashiwagi

**Contents**

**Descriptive Rules for Achalasia of the Esophagus**

I. Achalasia of the Esophagus
  1. Definition
  2. Symptoms
  3. Diagnosis
    3.1 Diagnostic features on esophagography
    3.2 Diagnostic features on upper gastrointestinal endoscopy
    3.3 Diagnostic features on esophageal manometry
  4. Classification
    4.1 Radiographic images of the esophagus
    4.2 Endoscopy
    4.3 Esophageal manometry
    4.4 Histopathology
  5. Treatment
    5.1 Pharmacotherapy
    5.2 Endoscopic treatment
    5.3 Surgical treatment
II. Other Esophageal Motility Disorders
  1. Diffuse esophageal spasm (DES)
  2. Nutcracker esophagus
  3. Hypertensive LES
  4. Non-specific esophageal motility disorder (NEMD)
  5. Esophageal motility dysfunctions complicating other diseases
  6. Pseudoachalasia

**I Achalasia of the esophagus**

**1. Definition**

Achalasia of the esophagus is an esophageal motility disorder of unknown etiology, characterized by failure of relaxation of the lower esophageal sphincter (LES) and impaired peristaltic movement of the lower esophageal body.

**2. Symptoms**

Symptoms include dysphagia, regurgitation of ingested food into the oral cavity, chest pain, weight loss, and nocturnal cough.

**3. Diagnosis**

Useful diagnostic modalities for this disorder include: (1) esophagography; (2) upper gastrointestinal endoscopy; (3) esophageal manometry; (4) histopathologic examination of the esophageal muscle layers (in patients undergoing surgery).

**3.1 Diagnostic features on esophagography**

1. Dilatation/tortuosity of the esophagus
2. Retained food in the esophagus and poor emptying of barium
3. Smooth conical narrowing of the esophagogastric junction (bird-beak sign)
4. Absence or diminution of gastric air bubbles
5. Abnormal esophageal motility

**3.2 Diagnostic features on upper gastrointestinal endoscopy**

1. Dilatation of the esophageal lumen
2. Abnormal retention of food and/or liquid remnants in the esophagus
3. Whitish change and thickening of the esophageal mucosal surface
4. Functional stenosis of the esophagogastric junction (endoscope passes through the stenotic segment although the esophagogastric junction fails to be dilated by insufflation; the procedure may involve winding of tissue around the scope or leafing of tissue on scope rotation)
5. Abnormal contraction waves of the esophagus

**3.3 Diagnostic features on esophageal manometry**

1. Deglutitive dysrelaxation of the LES^*^
2. Disappearance of primary peristaltic waves^*^
3. Increased esophageal static pressure (higher than the intragastric pressure)
4. Increased LES pressure
5. Occurrence of simultaneous contraction waves

^*^Principal finding

**4. Classification**

**4.1 Radiographic images of the esophagus**

X-ray examination: a dorsoventral view is obtained with the patient in the standing position; the patient is asked to swallow a barium meal, consisting of 100 mL of 100% barium sulfate, as quickly as possible, so that radiograms can be obtained within 1 min of the swallowing. No spasmolytic agent is used.

1. Morphologic type

**Fig. 1:**
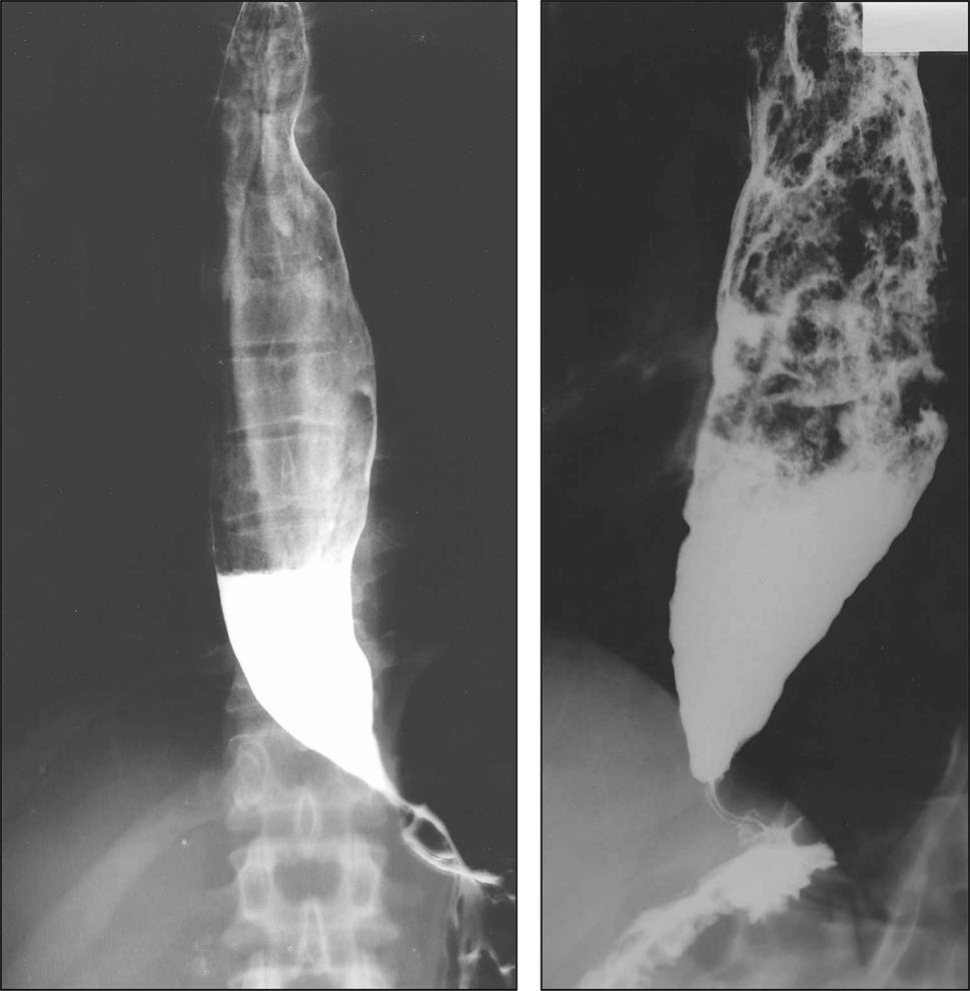
Straight (St) type
  Modest degree of tortuosity of the longitudinal axis of the esophagus. This type includes the conventional spindle type and flask type mentioned above.
ii. Sigmoid (Sg) type (Fig. 2)

This classification is based on the radiographic features of the esophagus. This classification, subclassified into the spindle type, flask type and sigmoid type according to the conventional descriptive rules, has been extensively used over decades. However, there are no distinct pathophysiological differences between the spindle type and the flask type, and no distinction is made between these two types in the United States or Europe. As for the sigmoid type, the extent of deviation and tortuosity of the upper esophagus may also have an impact on the therapeutic outcome. Therefore, achalasia is classified into the following two types in this version of the descriptive rules.

i. Straight (St) type (Fig. 1)

**Fig. 2:**
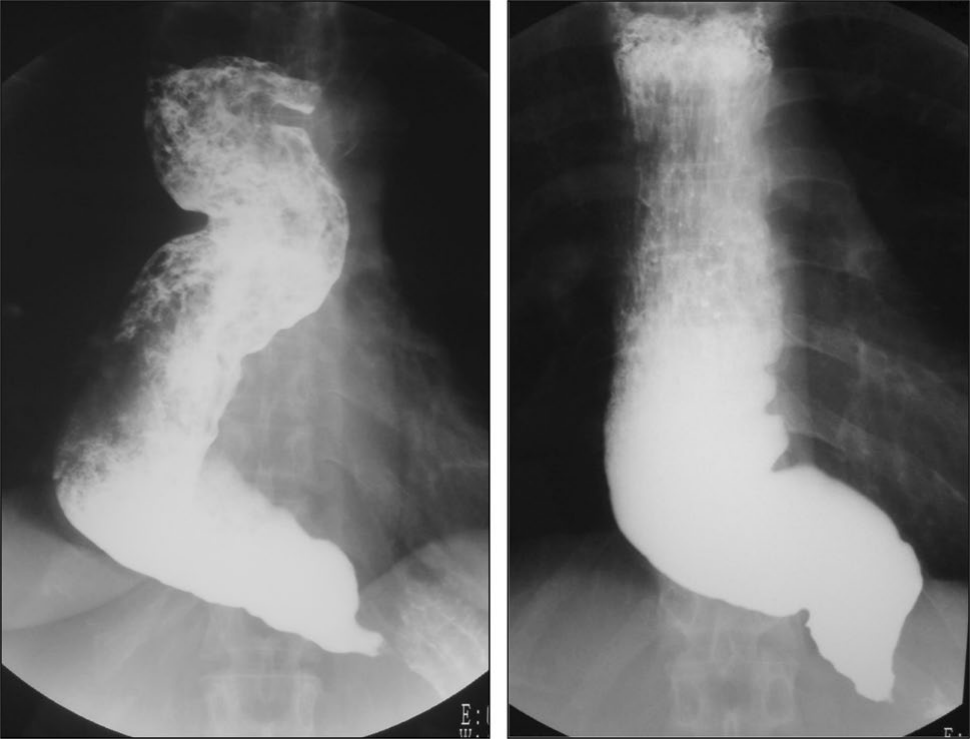
Sigmoid (Sg) type
  Significant degree of tortuosity of the longitudinal axis of the esophagus. Pronounced rightward tortuosity of the esophagus, causing the organ to assume an L-shaped course, is specifically termed the advanced sigmoid (aSg) type (Fig. 3).

**Fig. 3:**
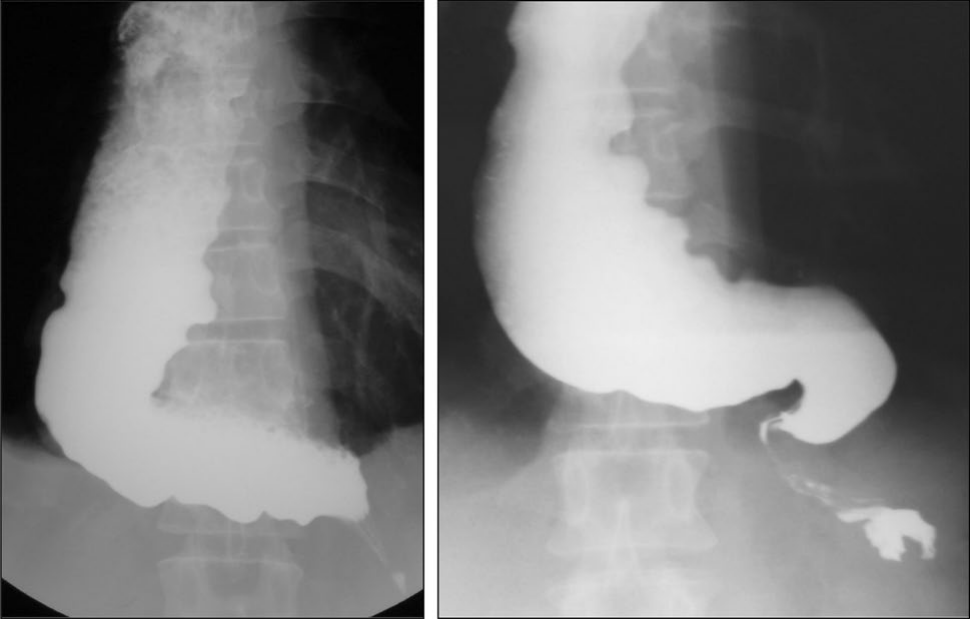
Advanced sigmoid (aSg) type

**Fig. 4:**
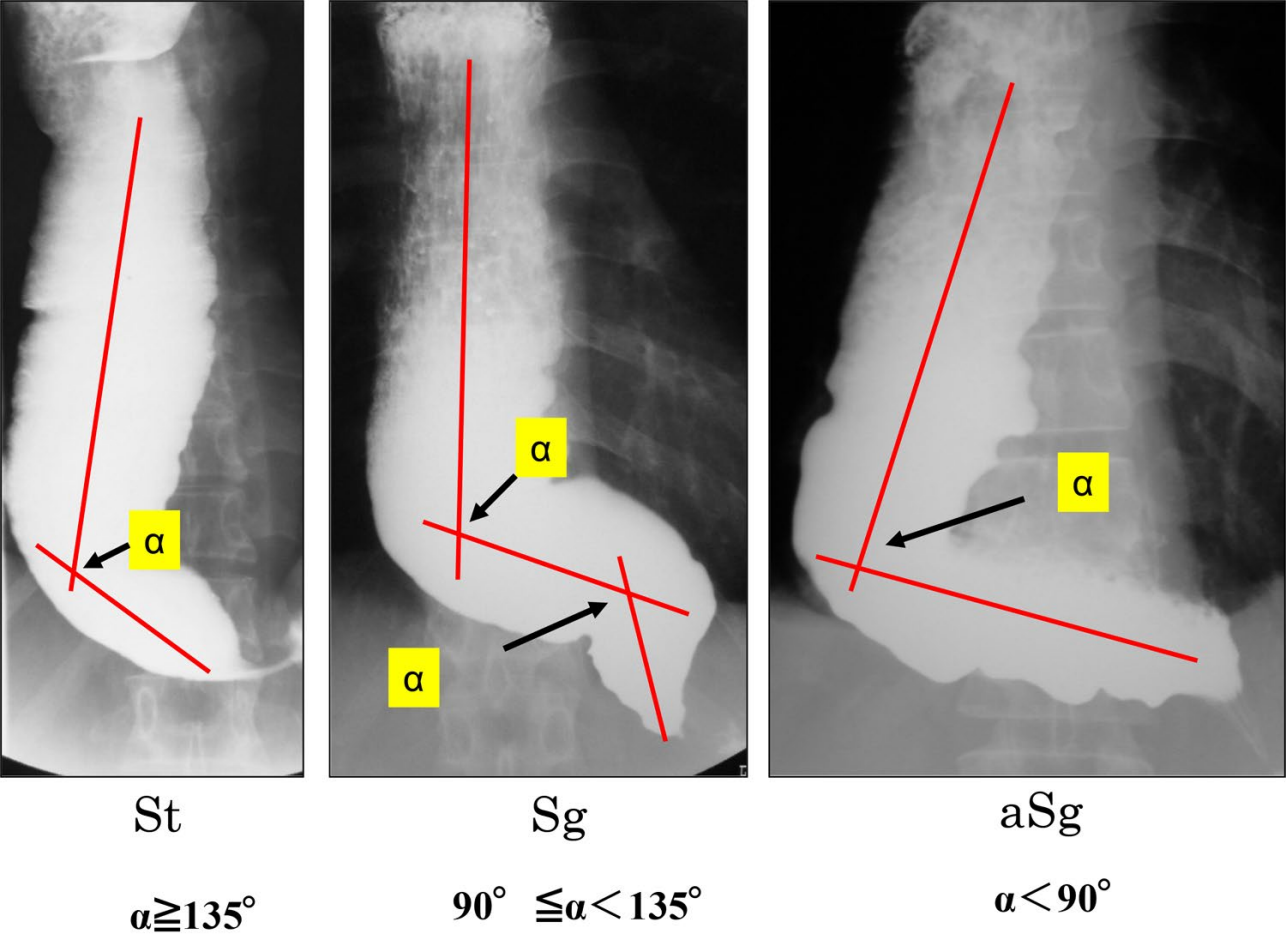
Distinction among the St, Sg, and aSg types

Addendum:

The St type, Sg type, and aSg type are illustrated in Fig. 4.

In cases where esophageal flexion(s) is noted, a straight line should be drawn in the direction of the esophageal long axis.

In case two straight lines are drawn, a single angle will be formed by the crossing straight lines; in case three straight lines are drawn, two angles will be formed by the crossing straight lines.

The following definitions are framed in order of increasing angle, *α*, formed by the crossing straight lines.

When *α* ≥ 135°, the disorder is diagnosed as the St type.

When 90° ≤ *α* < 135°, the disorder is diagnosed as the Sg type.

When *α* < 90°, the disorder is diagnosed as the aSg type.

The angle(s) should be stated without fail.

**Fig. 5:**
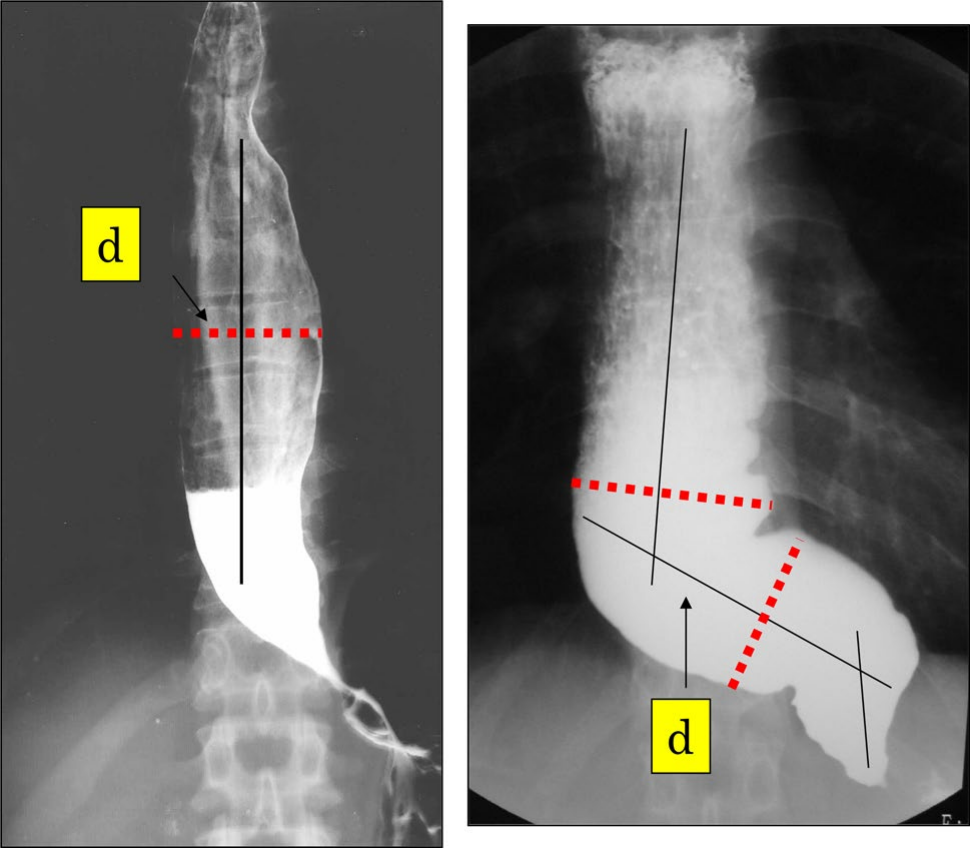
Dilatation grading. Grade I d < 3.5 cm, Grade II 3.5 ≤ d < 6.0 cm, Grade III 6.0 cm ≤ d

(2) Dilatation grading (Fig. 5)

The dilatation is classified into Grades I to III according to the maximum transverse diameter (*d*) of the esophagus drawn perpendicularly to the longitudinal axis.

i. Grade I *d* < 3.5 cm
ii. Grade II 3.5 ≤ *d* < 6.0 cm
iii. Grade III 6.0 cm ≤ *d*

**Fig. 6:**
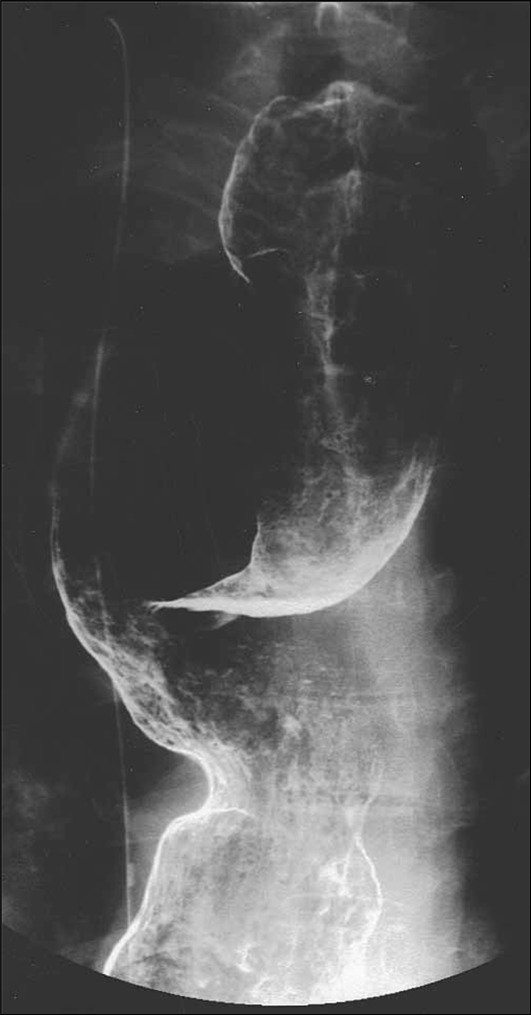
Tortuosity of the upper thoracic esophagus
ii. Leftward-tortuous esophagus (Fig. 7)

Addenda:

i. Tortuosity of the upper thoracic esophagus (Fig. 6)

**Fig. 7:**
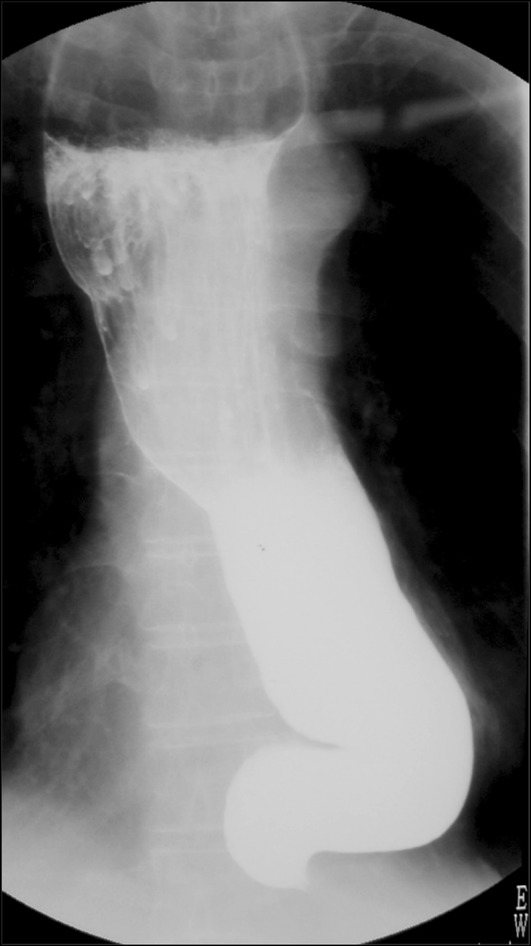
Leftward tortuosity of the esophagus
iii. Concurrent epiphrenic diverticulum (pulsion diverticulum) (Fig. 8)

**Fig. 8:**
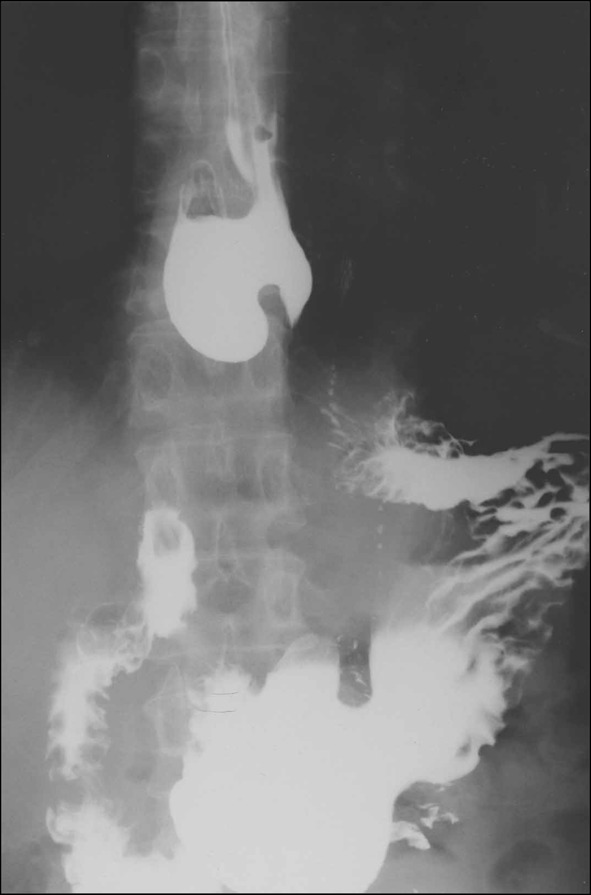
Concurrent epiphrenic diverticulum
iv. Concurrent carcinoma of the esophagus (location, size, and depth of invasion should be stated)

**Fig. 9:**
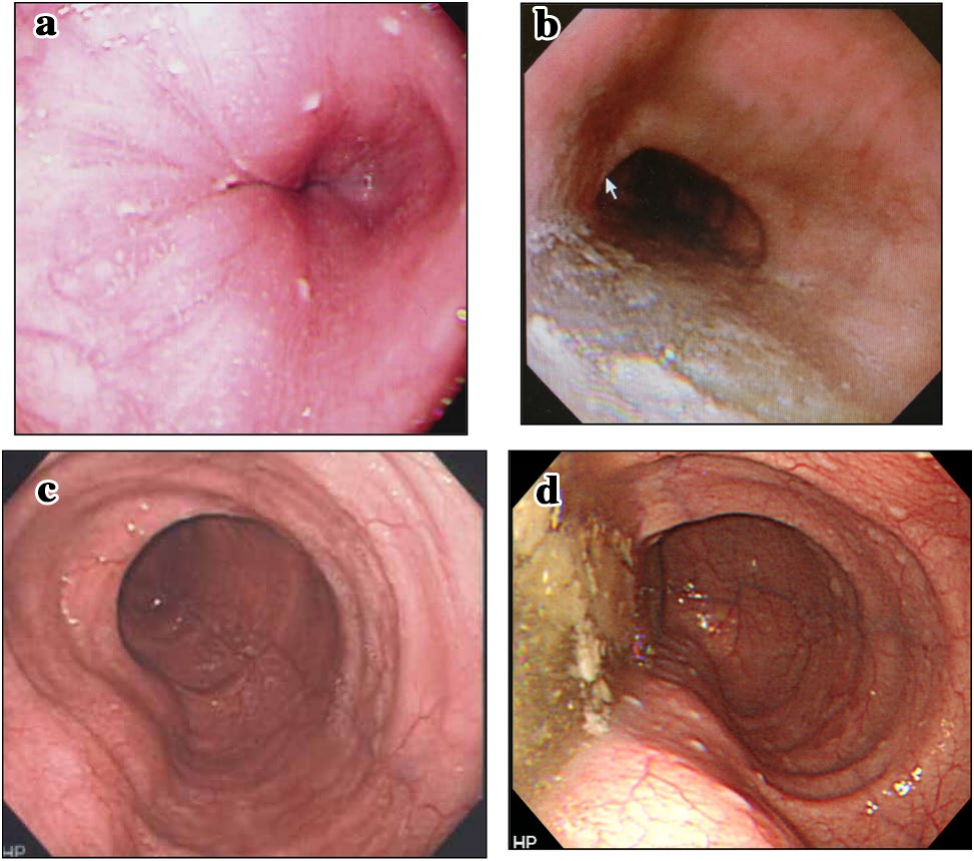
Endoscopic images. Normal type (**a**), retained type (**b**), dilated type (**c**), and dilated-retained type (**d**)

**4.2 Endoscopy** (Fig. 9)

Achalasia of the esophagus is classified into the following four types according to the endoscopic findings.

i. Normal type: there is no obvious retention of ingested food or dilatation of the esophagus.
ii. Retained type: retention of ingested food or fluid in the esophagus, but no apparent dilatation.
iii. Dilated type: dilatation of the esophagus is evident, but no retained ingested food is seen in the esophagus.
iv. Retained-dilated type: the esophagus is dilated with retained ingested food.

**Fig. 10:**
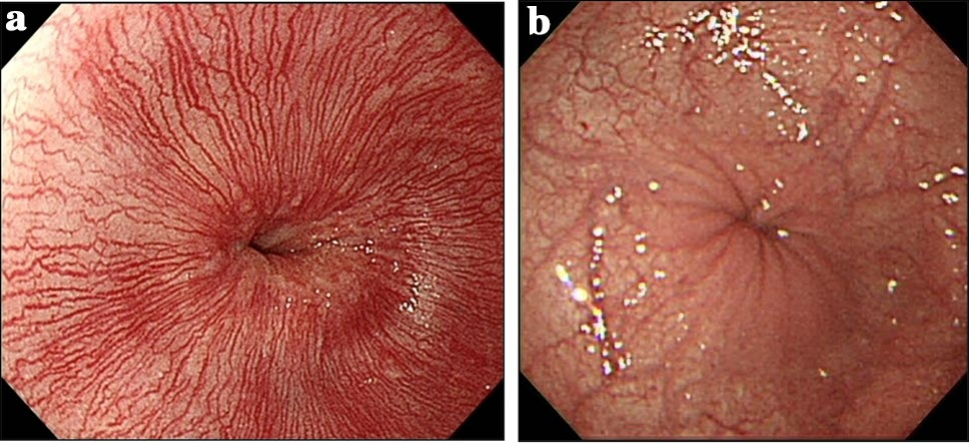
Endoscopic images of the cardiac portion of the esophagus. Healthy subject (**a**) and a patient with achalasia (**b**)

Reference findings suggestive of achalasia (Fig. 10)

In healthy subjects who inspire deeply, the lower esophagus usually opens, and an entire view of the esophageal palisade vessels becomes visible. However, in patients with achalasia, an entire view of the esophageal palisade vessels does not become visible and, in addition, rosette-like esophageal folds appear in the lower esophagus [1].

**4.3 Esophageal manometry**

Achalasia of the esophagus is classified into the following two types according to the findings on esophageal manometry.

i. Complete type
  Impaired relaxation of the LES and the absence of esophageal peristalsis in response to swallowing.
ii. Incomplete type
  Not meeting the above criteria.

**Fig. 11:**
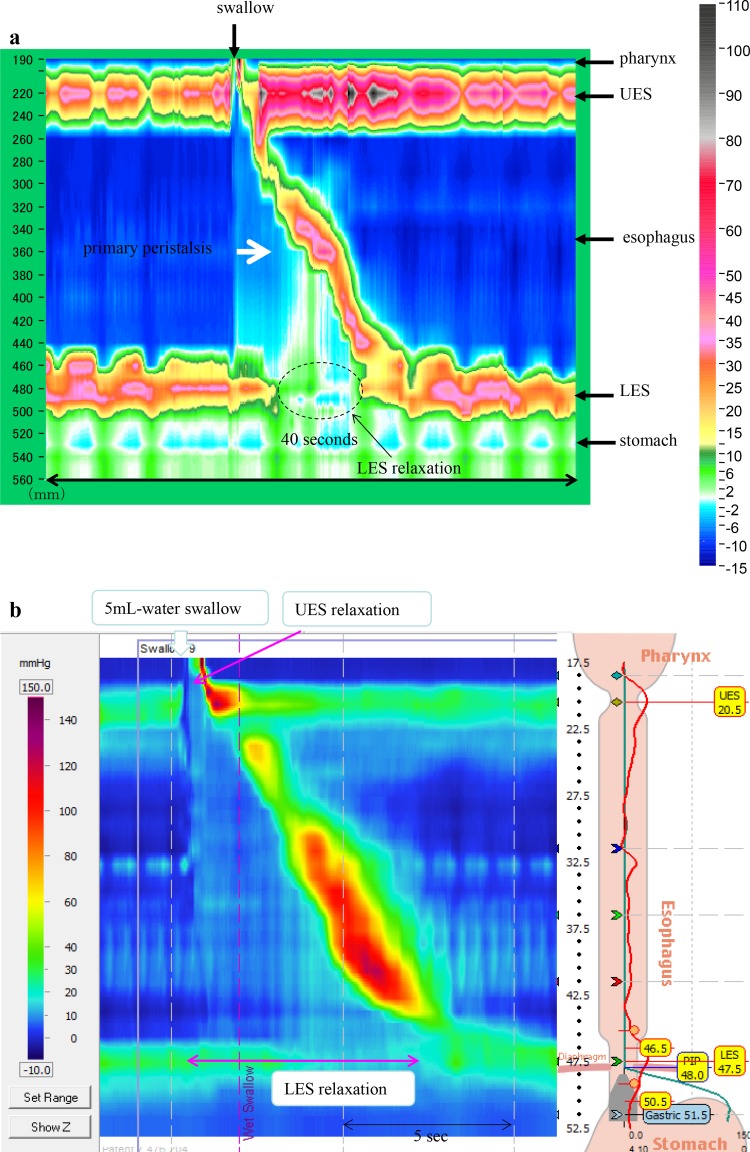
Esophageal high-resolution manometry findings of primary peristalsis and lower esophageal sphincter (LES) relaxation after water swallowing in healthy subject.** a** Manometric instrument: Trace! (Dr. G.S. Hebbard, The Royal Melbourne Hospital, Parkville, Australia).** b** Manometric instrument: ManoScan 360™ (Sierra Scientific Instruments Inc.)

**Fig. 12:**
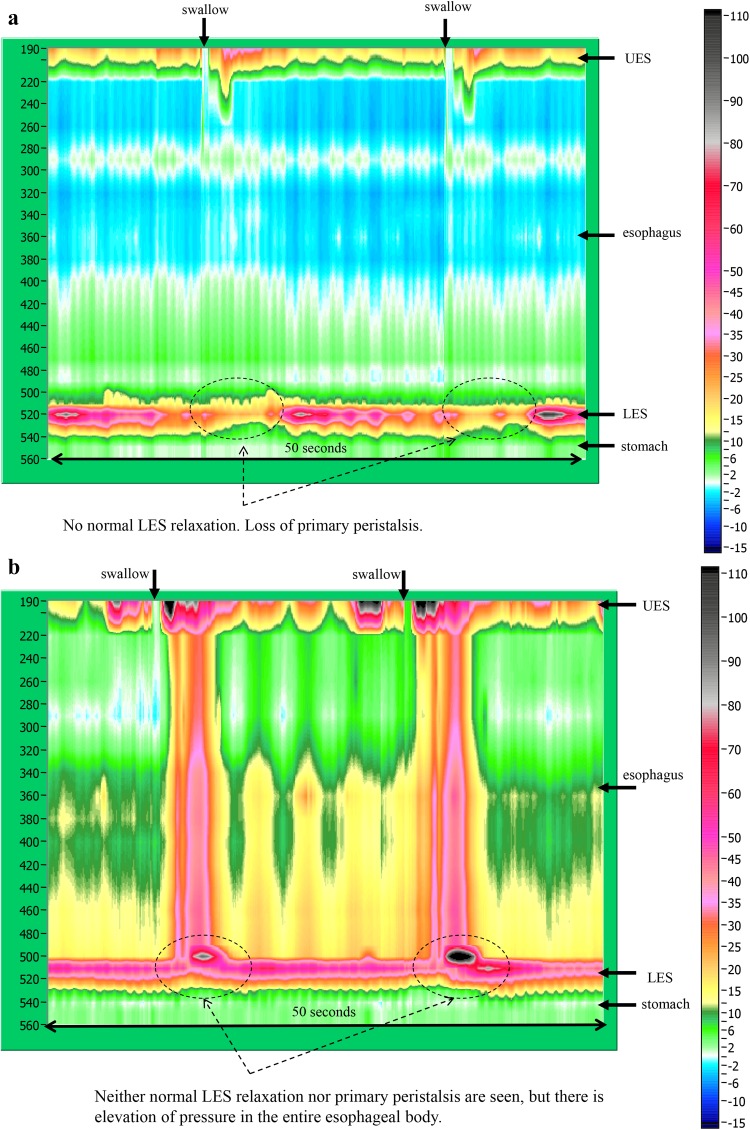
Esophageal high-resolution manometry findings in a patient with achalasia. Manometric instrument: Trace! (Dr. G.S. Hebbard, The Royal Melbourne Hospital, Parkville, Australia)

Esophageal manometric findings in healthy subjects (Fig. 11a, b)

Esophageal manometric findings in patients with achalasia (Fig. 12, b)

1. Impaired relaxation of the LES
2. Absence of esophageal peristalsis

**Fig. 13:**
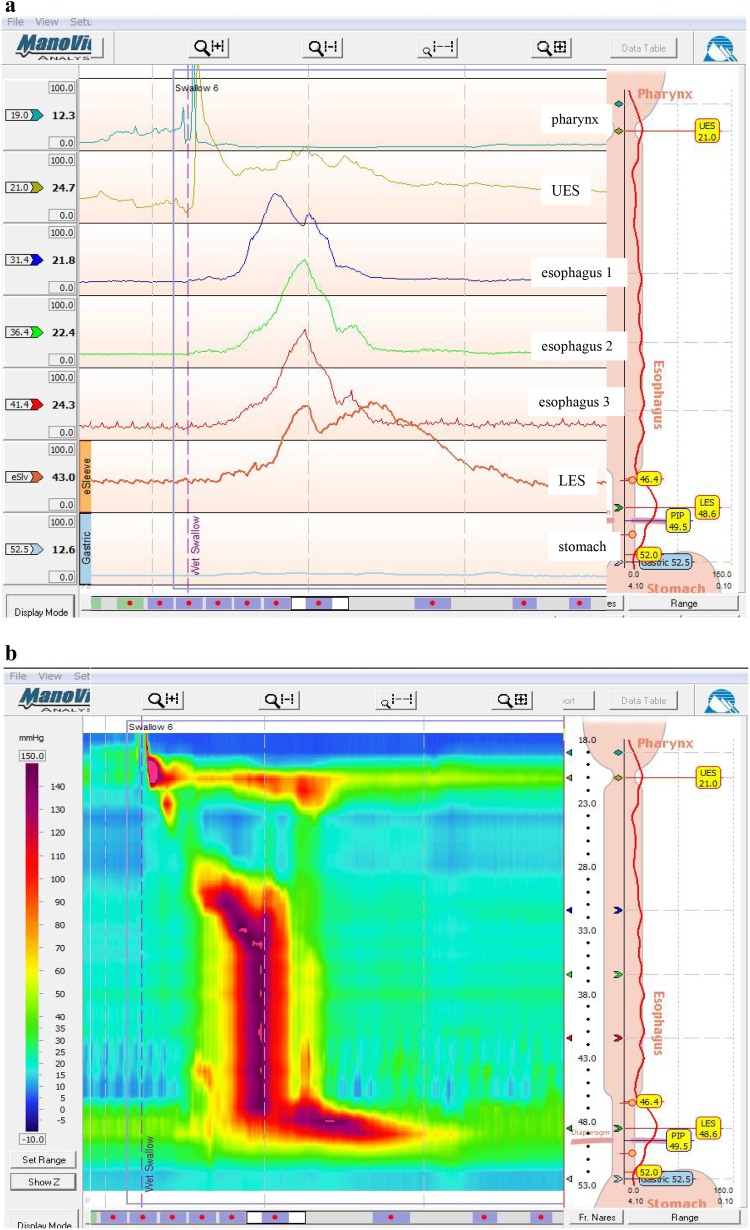
Esophageal manometry findings in a patient with vigorous-type achalasia. Manometric instrument: ManoScan 360™ (Sierra Scientific Instruments Inc.).** a** Conventional method.** b** Esophageal high-resolution manometry

Addendum:

i) Vigorous achalasia [2] (Fig. 13a, b)

In addition to the above diagnostic criteria, when amplitude of the lower esophagus is ≥37 (30 to 40) mmHg, this condition is defined as vigorous achalasia, and when amplitude of the lower esophagus is <37 (30 to 40) mmHg, it is termed classic achalasia.

**Fig. 14:**
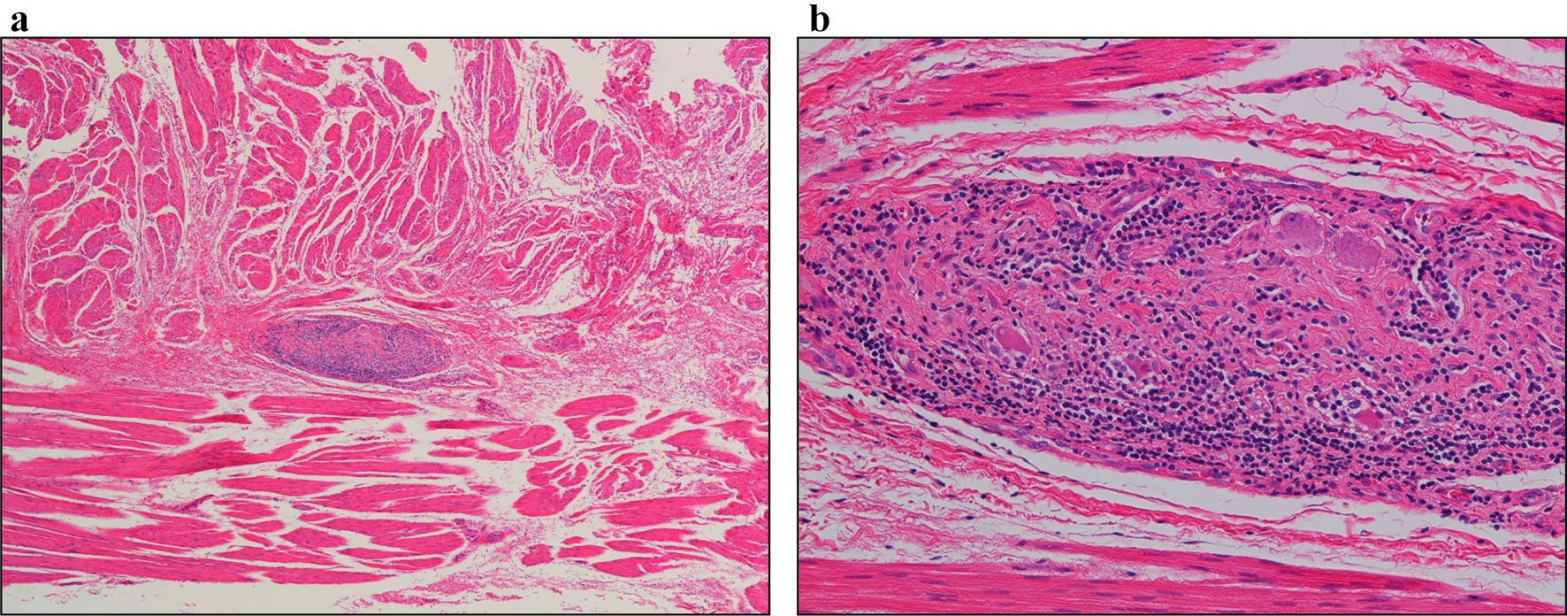
Histopathologic photomicrographs (Grade II).** a** Lymphocyte accumulation in Auerbach’s plexus between the hypertrophic inner circular and outer longitudinal muscle layers.** b** Close-up view of a. Prominent lymphocyte infiltration and fibrosis are evident in Auerbach’s plexus. There is an evident decrease in the number of neuroganglion cells and degeneration of neuroganglion cells

**Fig. 15:**
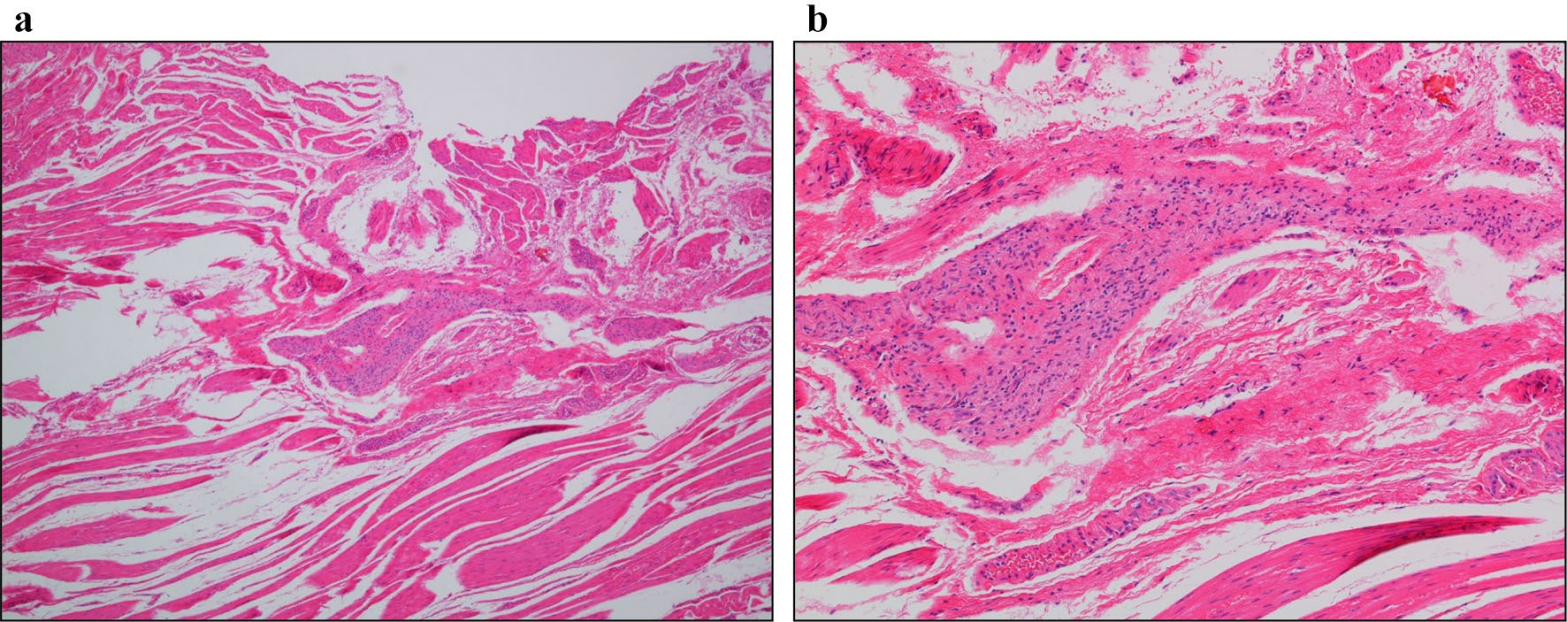
Histopathologic photomicrographs (Grade III).** a** A smaller Auerbach’s plexus is evident in the hypertrophic inner circular and outer longitudinal layers of the tunica muscularis propria.** b** Close-up view of a. Fibrosis is evident in Auerbach’s plexus. Loss of neuroganglion cells and the nerve fiber network is observed

**4.4 Histopathology** (Figs. 14, 15)

It is advisable for evaluation of the esophageal muscularis propria to be performed both on specimens from the dilated segment and narrowed segment. The tissue sampling sites must be stated. Achalasia of the esophagus is classified under the following three categories according to the ganglion cell/nerve fiber status observed on histopathology.

1. Grade I: practically normal ganglion cells and nerve fibers.
2. Grade II: diminution and degeneration of ganglion cells and nerve fibers.
3. Grade III: loss of ganglion cells and nerve fibers.

Addendum: note if there is any lymphocytic infiltration, etc.

**5. Treatment**

**5.1 Pharmacotherapy **[3–10]

Calcium channel antagonists (e.g., Adalat^®^ Capsule) and nitrates (e.g., Nitorol^®^ Tablet) are used as drugs to relax the LES. In patients with pronounced subjective symptoms, complete relief of the symptoms can often not be obtained with pharmacotherapy alone. Currently, pharmacotherapy is generally recognized as the treatment option of choice for patients with mild disorder and for those who are not suitable candidates for endoscopic therapy or surgery, as well as for temporary relief of symptoms. The medications are also prescribed as needed in case of postoperative chest pain. Calcium antagonists are subject to off-label use in terms of national health insurance scheme.

**5.2 Endoscopic treatment**

(1) Dilatation therapy [11–13]

Following endoscopic insertion of a guidewire into the stomach, a balloon (usually, a 30-mm balloon) is placed at the gastroesophageal junction along the guidewire under fluoroscopic guidance; the balloon is then gradually inflated, beginning at a low pneumatic pressure level, while checking for pain until the balloon notches disappear. The pneumatic pressure is further elevated slightly to dilate the balloon while checking for pain, although excessive pressurization may be risky. The reported effectiveness rates of balloon dilatation range from 66 to 93%, hence virtually comparable to effectiveness rates of surgical treatment. However, the reported effectiveness rates of balloon dilatation in young adults (under 40 years of age) are poorly responsive to treatment. Symptoms recur in about 33% of patients at 4 to 6 years after balloon dilatation. Complications include reflux esophagitis, chest pain, fever, and perforation.

The conditions and frequency of balloon dilatation should be stated in each case of dilatation therapy.

(2) Botulinum toxin local injection therapy [14, 15]

Botulinum toxin local injection reduces in the LES pressure via inhibiting neurotransmitter release from the preganglionic cholinergic neurons. In botulinum toxin local injection therapy, 100 IU of type A botulinum toxin is injected endoscopically into the 4 quadrants of the LES. Relapse is often reported in 6–12 months. Use of botulinum toxin local injection therapy for achalasia cardia is subject to off-label use for the purpose of coverage by the national health insurance scheme in Japan.

(3) Endoscopic myotomy [16]

This procedure consists of peroral endoscopic myotomy performed on the esophageal submucosa. Use of endoscopic myotomy for the treatment of achalasia cardia is listed as off-label use for the purpose of coverage by the national health insurance scheme.

**5.3 Surgical treatment** [17–24]

Surgical treatment [17–24]

Surgical treatment was previously performed via a laparotomy, and the procedure has recently become supplanted by a laparoscopic technique. Heller’s myotomy to relieve obstruction combined with a reflux-preventive procedure to prevent post-myotomy gastroesophageal reflux, especially the Dor technique, is widely used, with gratifying response rates of as high as about 90%.

The length of muscle layer incision, whether the short gastric arteries vessels were dissected or not, whether an esophageal bougie was inserted during the operation or not, and whether there was any intraoperative mucosal injury or not should be stated.

**II Other Esophageal Motility Disorders** [25−30]

**Fig. 16:**
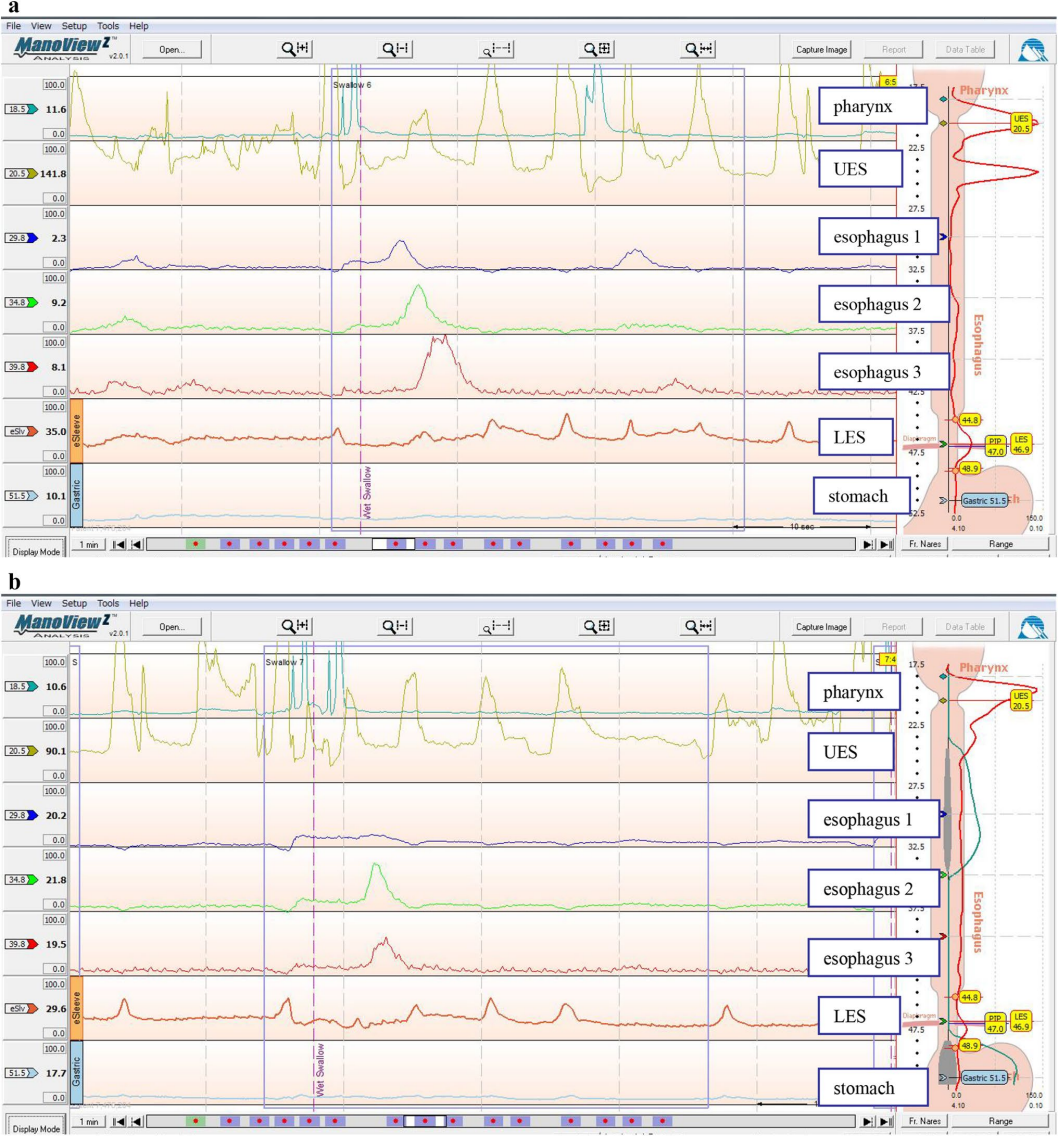
Esophageal manometry in a patient with DES by ManoScan 360™ (Sierra Scientific Instruments Inc.).** a** Normal peristalsis.** b** Simultaneous contractions

**Fig. 17:**
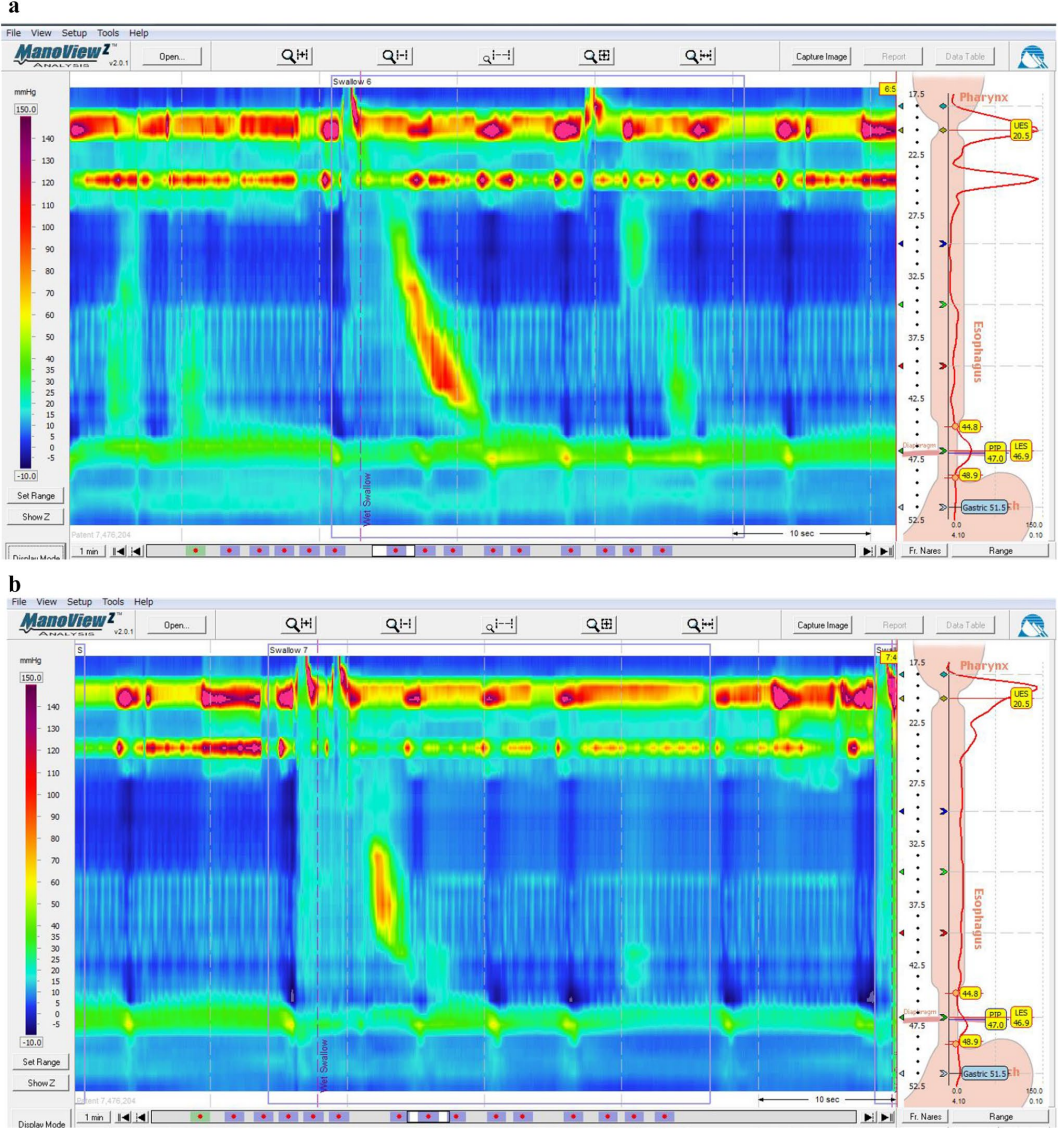
Esophageal manometry in a patient with DES by high-resolution manometry.** a** Normal peristalsis.** b** Simultaneous contractions

**Fig. 18:**
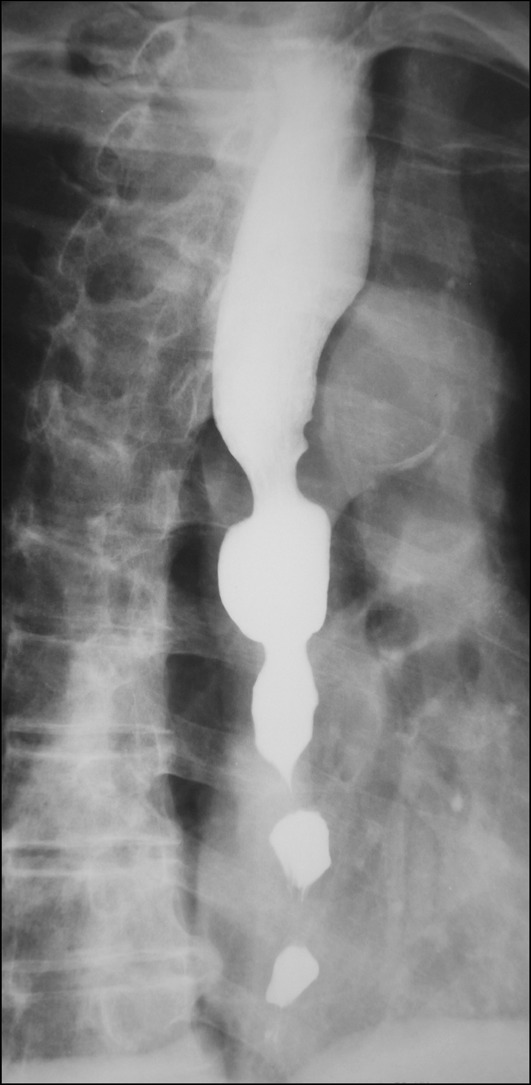
Radiographic appearance in a patient with DES

**1. Diffuse esophageal spasm (DES)** (Figs. 16, 17, 18)

DES is a motility disorder characterized by occasional, persistent or repetitive abnormal contractions while peristaltic waves are usually normal, and may cause dysphagia and chest pain. One of the diagnostic criteria is that 2 or more repetitive simultaneous contractions account for 20% or more of the peristalsis on water swallow. The abnormality may be accompanied with multi-peaked contractions, elevation of the amplitude of the peristaltic waves, spontaneous contractions, and incomplete LES relaxation. Esophageal X-ray findings may include simultaneous contractions (nonperistaltic contractions), corkscrew appearance, arrest of contrast medium passage, and pocket formation.

**Fig. 19:**
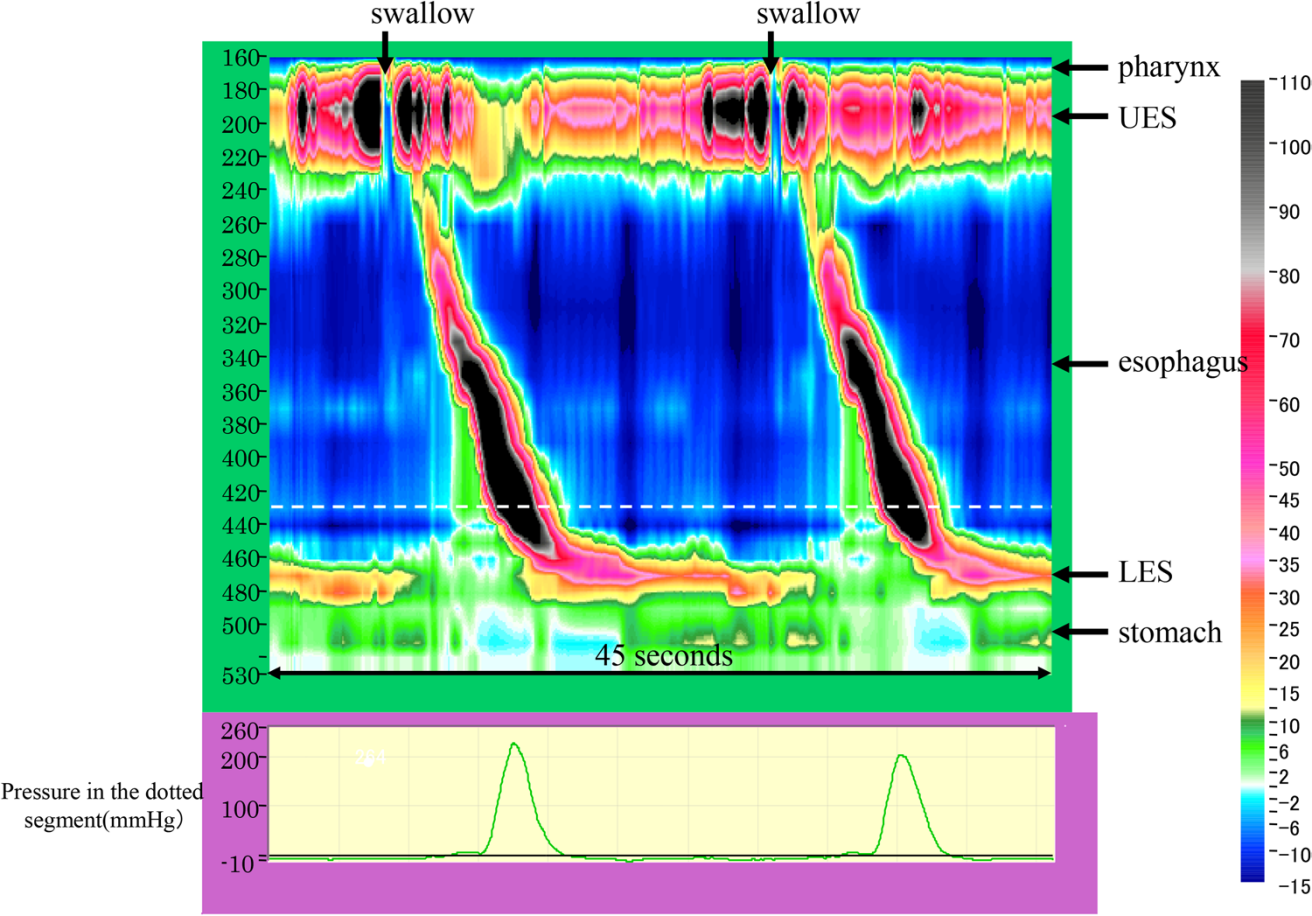
Esophageal high-resolution manometry findings in a patient with nutcracker esophagus. Manometric instrument: Trace ! (Dr. G.S. Hebbard, The Royal Melbourne Hospital, Parkville, Australia)

**Fig. 20:**
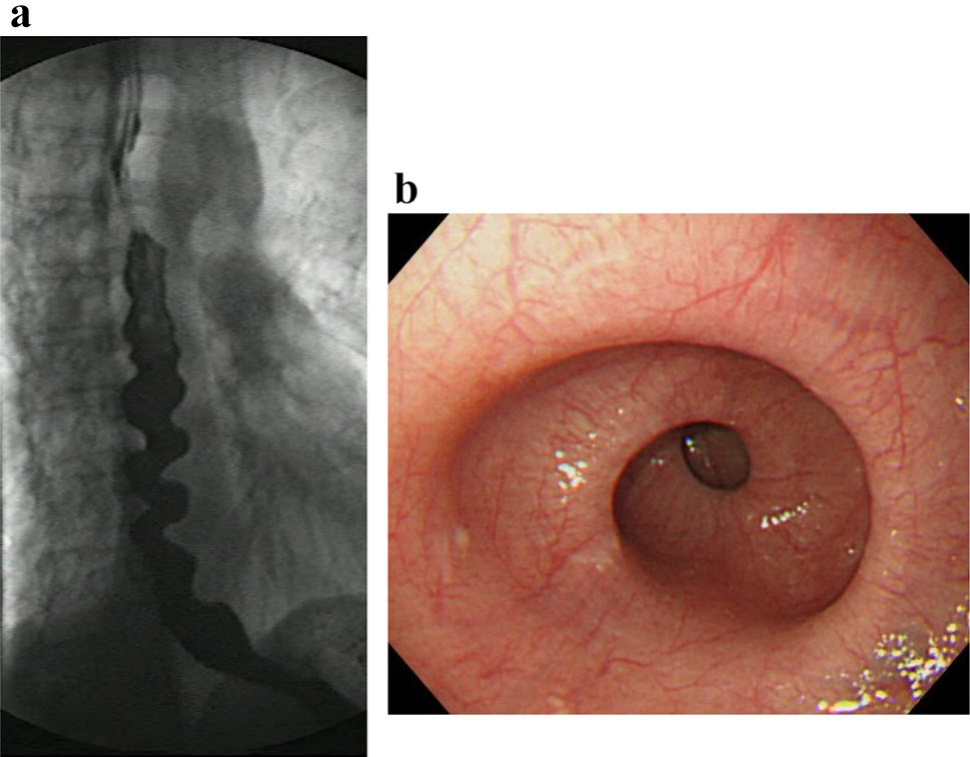
X-ray (**a**) and endoscopic (**b**) images of nutcracker esophagus

**2. Nutcracker esophagus **(Figs. 19, 20)

Nutcracker esophagus is one of primary esophageal motility disorders. Nutcracker esophagus is defined as normal peristalsis of the esophageal body with an average distal esophageal amplitude exceeding 180 mm Hg and often has non-cardiac chest pain or dysphagia. Barium swallows are usually normal but a corkscrew-like esophagus, reminiscent of the findings of diffuse esophageal spasm (DES), is observed occasionally.

**3. Hypertensive lower esophageal sphincter (Hypertensive LES)**

This esophageal motility disorder is characterized by elevation of LES pressure, causing chest pain and dysphagia. A mean resting LES static pressure of ≥45 mmHg, normal relaxation of the LES, and normal peristaltic waves of the esophageal body are essential for diagnosis of this disorder.

**Fig. 21:**
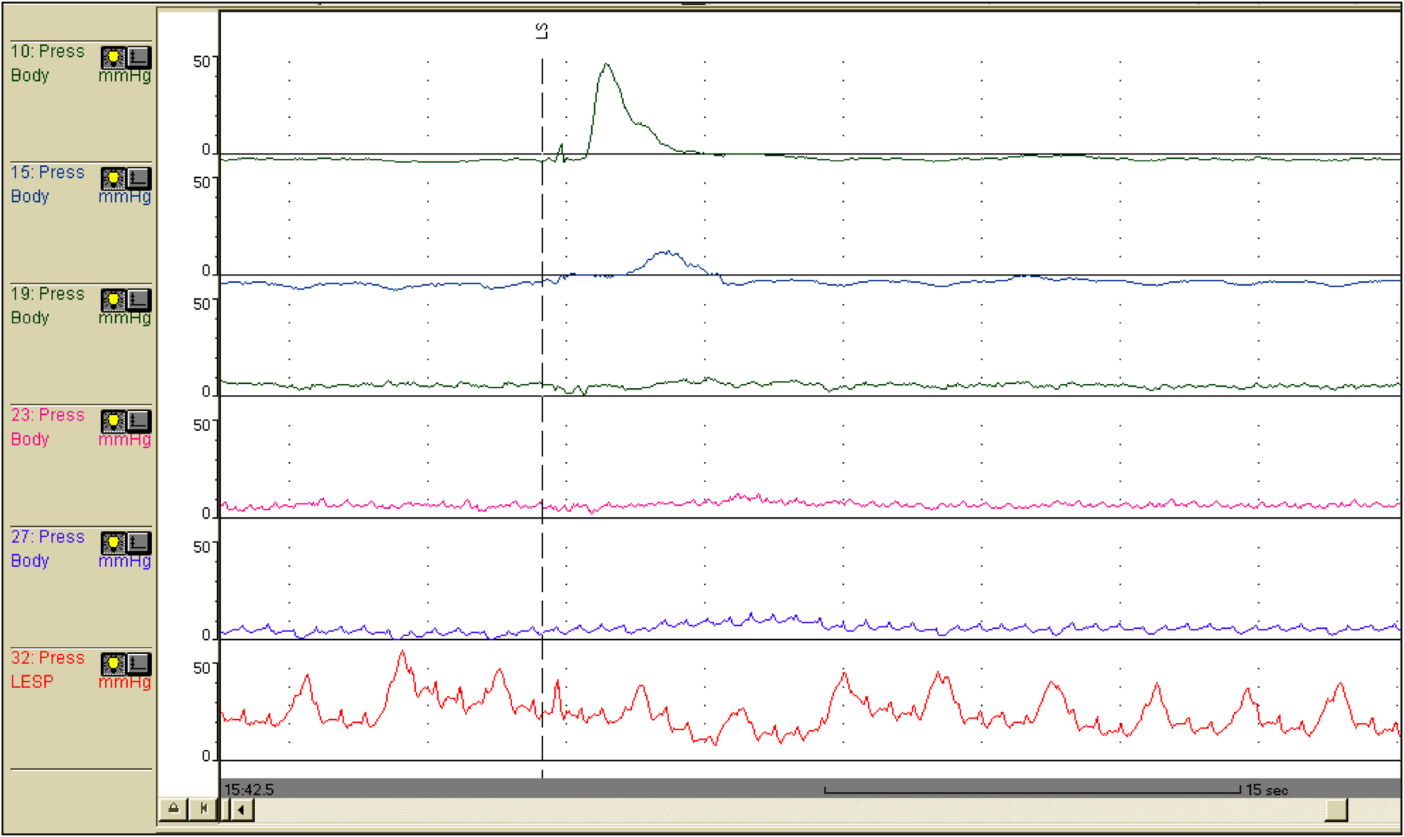
Esophageal manometric features in NEMD. Manometric instrument: INSIGHT (Sandhill Scientific Instruments Inc.)

**4. Non-specific esophageal motility disorder (NEMD) (Fig.** 21)

NEMD represents a spectrum of esophageal motility abnormalities presenting with apparent esophageal manometric tests, but failing to meet the diagnostic criteria for the esophageal manometric features of achalasia, DES, nutcracker esophagus or hypertensive LES. The most common manometric finding among the esophageal motility abnormalities is the occurrence of low contraction waves with amplitudes of ≤30 mmHg or nonpropagating contractions in the lower esophagus. According to the latest classification of esophageal manometry, detection of the above manometric finding on water swallowing at a frequency of ≥30% is newly defined as ineffective esophageal motility (IEM).

**5. Esophageal motility dysfunctions complicating other diseases**

Esophageal motility dysfunction may be associated with collagen diseases such as scleroderma and systemic lupus erythematosus, diabetes mellitus, amyloidosis, eosinophilic esophagitis, neurodegenerative disorders such as Parkinson’s disease, and alcohol dependence.

**6. Pseudoachalasia**

This disorder presents with apparently achalasia-like symptoms/findings associated with malignancy, such as carcinoma of the esophagogastric junction.
